# Sulfur Cycling as a Viable Metabolism under Simulated Noachian/Hesperian Chemistries

**DOI:** 10.3390/life12040523

**Published:** 2022-04-01

**Authors:** James A. W. Oliver, Matthew Kelbrick, Nisha K. Ramkissoon, Amy Dugdale, Ben P. Stephens, Ezgi Kucukkilic-Stephens, Mark G. Fox-Powell, Susanne P. Schwenzer, André Antunes, Michael C. Macey

**Affiliations:** 1 Biology Department, Edge Hill University, Ormskirk L39 4QP, UK; 23187824@edgehill.ac.uk (J.A.W.O.); matthew.kelbrick@liverpool.ac.uk (M.K.); 2 Department of Evolution, Ecology and Behaviour, Institute of Infection, Veterinary and Ecological Sciences, University of Liverpool, Liverpool L69 3GJ, UK; 3 AstrobiologyOU, School of Environment, Earth and Ecosystem Sciences, Faculty of Science, Technology, Engineering and Mathematics, The Open University, Milton Keynes MK7 6AA, UK; nisha.ramkissoon@open.ac.uk (N.K.R.); ben.stephens@open.ac.uk (B.P.S.); ezgi.kucukkilic-stephens@open.ac.uk (E.K.-S.); mark.fox-powell@open.ac.uk (M.G.F.-P.); susanne.schwenzer@open.ac.uk (S.P.S.); 4 AstrobiologyOU, School of Physical Sciences, Faculty of Science, Technology, Engineering and Mathematics, The Open University, Milton Keynes MK7 6AA, UK; amy.dugdale@open.ac.uk; 5 Biology Department, Maynooth University, Maynooth, W23 F2H6 Kildare, Ireland; 6 School of Earth & Environmental Sciences, University of St Andrews, Fife KY16 9AJ, UK; 7 State Key Laboratory of Lunar and Planetary Sciences, Macau University of Science and Technology (MUST), Macau, China; aglantunes@must.edu.mo; 8 China National Space Administration (CNSA), Macau Center for Space Exploration and Science, Macau, China

**Keywords:** simulation, analogue, sulfur, enrichment

## Abstract

Water present on the surface of early Mars (>3.0 Ga) may have been habitable. Characterising analogue environments and investigating the aspects of their microbiome best suited for growth under simulated martian chemical conditions is key to understanding potential habitability. Experiments were conducted to investigate the viability of microbes from a Mars analogue environment, Colour Peak Springs (Axel Heiberg Island, Canadian High Arctic), under simulated martian chemistries. The fluid was designed to emulate waters thought to be typical of the late Noachian, in combination with regolith simulant material based on two distinct martian geologies. These experiments were performed with a microbial community from Colour Peak Springs sediment. The impact on the microbes was assessed by cell counting and 16S rRNA gene amplicon sequencing. Changes in fluid chemistries were tested using ICP-OES. Both chemistries were shown to be habitable, with growth in both chemistries. Microbial communities exhibited distinct growth dynamics and taxonomic composition, comprised of sulfur-cycling bacteria, represented by either sulfate-reducing or sulfur-oxidising bacteria, and additional heterotrophic halophiles. Our data support the identification of Colour Peak Springs as an analogue for former martian environments, with a specific subsection of the biota able to survive under more accurate proxies for martian chemistries.

## 1. Introduction

Water is essential to life, thus limiting the potential time frames and geographical locations of Mars where life could exist. In general, water’s existence is confined to earlier periods in Mars’ history, during the Noachian period (4.0–3.7 Gya), or in the modern subsurface, persisting either as groundwater or in regions of high geothermal heat flux [1,2,3]. The historical presence of water on Mars is evidenced by geomorphological features such as streambeds [3] and water–rock interactions documented by minerals that have undergone hydrous alteration [4]. These aqueous environments would have dried out episodically and later permanently (during the transition from the Noachian to the arid Hesperian era, i.e., 3.7–3.0 Gya) due to the loss of atmosphere that occurred towards the end of the Noachian [5]. This atmospheric shift would have caused waters to evaporate, resulting in increasingly concentrated fluids. 

The study of analogue environments can inform the development of hypotheses with regards to the potential habitability of the water of Mars and the viability of specific metabolic processes. One such analogue environment is Colour Peak. Colour Peak is a sulfidic and saline spring system on Axel Heiberg Island in the Canadian High Arctic, the chemistry and microbial community of which has been characterised in detail [6,7]. Colour Peak is considered an analogue for late-Noachian period Mars due to the similarities of the chemistry of its waters and the temperature regime to those proposed for the Noachian period (4.0 Gya) [7,8,9]. Prior work (Macey et al., 2020b) that investigated the potential viability of metabolisms under martian chemical conditions predicted that sulfur-based metabolisms (sulfur oxidation and sulfate reduction) would be viable. This prediction was based on the microbial community of this environment being dominated by sulfur-oxidising bacteria [7,10] and sulfur oxidation and sulfate reduction being the most thermodynamically productive metabolisms in modelled martian waters [7,11].

However, experimental testing of which microbes from analogue environments can persist in the simulated martian chemical environments expected for the Noachian–Hesperian transition is required to further develop ideas and challenge hypotheses concerning habitability and viability of former martian aqueous environments. Therefore, the main aim in this study was to test the predictions made in Macey et al., 2020, assessing the viability of the microbial community of sediments from Colour Peak Springs under simulated martian chemical environments. Given the potential for sulfur-cycling bacteria to produce biosignatures as a consequence of their metabolisms (e.g., the preservation of specific biological cofactors [12]), a greater understanding of their viability under simulated martian chemical conditions is necessary. 

The simulated chemical environments tested in this study were derived from thermochemical modelling of Gale Crater alteration mineral assemblages and represent water chemistries relevant to the Noachian–Hesperian transition, as early-stage sediment deposition in Gale Crater spans this time [13]. To enhance the chemical relevance of the simulation experiments and to ensure a supply of trace elements, two regolith simulants were also provided [14]. One of the simulants was based on the chemistry of the Rocknest site at Gale Crater [15], representative of the globally distributed dust, and the environment from which the Noachain–Hesperian (NH) simulated fluid was derived. The other regolith simulant chemistry was based on Haematite Slope, a locally Fe^3+^ enriched regolith [16]. This alternate regolith simulant was included to assess the relative importance of the regolith chemistry to habitability of the simulated chemistries and the viabilities of individual metabolisms. 

## 2. Materials and Methods

### 2.1. Sample Collection and Characterisation

**Figure 1:**
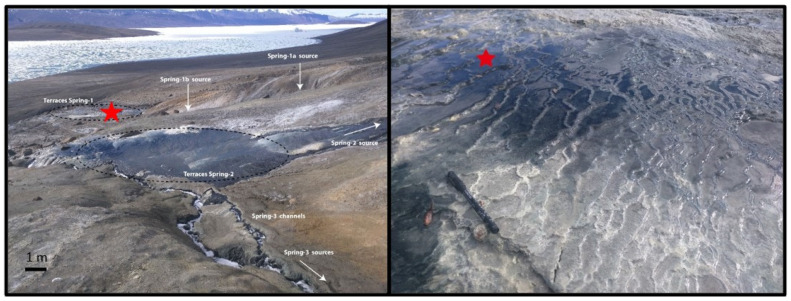
Photographs of Colour Peak Springs highlighting the sample collection point. These images are reproduced from [7] under creative commons licence 4.0 (creativecommons.org/licenses/by/4.0/).

Samples from the Colour Peak Springs were collected during the summer field season in 2017 as previously described in [6]. Sediments were collected aseptically from a sediment-rich pool (Figure 1) (79.3814°, −91.2727°), kept in a sealed bottle to exclude air, and stored at ambient arctic temperatures whilst in the field. These samples were transported to the Open University (United Kingdom) and stored at 4 °C. Samples were stored until culturing started in June 2019.

### 2.2. Preparation of Simulated Martian Fluids 

The fluids in this experimental series are referred to as NH fluids in the text. The NH fluids are established as appropriate for the Noachian–Hesperian transition as they are derived from the chemistries established in [17], using the program CHIM-XPT [18] to thermochemically model the interaction between 1 kg of water and 1 g of Rocknest soil and model the evaporation of these fluids to the sulfate forming stage to arrive at the mineralogy of Gale Crater evaporites and the corresponding solution chemistry during this point in the history of Mars. The elements that remained in solution were paired to produce a medium (Components in mM are: FeSO_4_ 1.29 × 10^−9^, MgSO_4_ 1.60 × 10^−5^, MnSO_4_ 0.0007, NaHS 0.0025, NaHCO_3_ 0.0318, K_2_SO_4_ 1.4246, Na_2_HPO_4_ 4.86 × 10^−9^, CaCl_2_ 0.8457, SiO_2_ 0.0714, NaCl 5.8911, NaOH 13.0825). The starting pH of the fluid was 9.5, which shifted to 7.28 and 9.78 following combination with simulant material based on either the chemistry of Rocknest at Gale Crater (OUCM-1—representative of a basaltic lithology) or Haematite Slope (OUHR-1—representing an Fe^3+^ rich lithology), respectively. The NH fluids were made in 1 L volumes and in an anaerobic chamber (Coy, Grass Lake, United States) with a headspace of CO_2_/H_2_/N_2_ (90:5:5) to prevent the oxidation of the individual components. The water used for these fluids was boiled to reduce the concentration of dissolved oxygen. The components comprising the fluids were prepared separately under anoxic conditions as above, and aliquots of these added individually to produce complete fluids of the desired elemental composition. 

### 2.3. Establishing Enrichment Series

In an anaerobic chamber (Coy, Grass Lake, MI, USA), 39 mL of the NH fluids was combined with 10 g of either OUCM-1 or OUHR-1 simulant in a 100 mL glass serum vial [14]. Ten grams of Colour Peak sediment was combined with 100 mL of NH fluid to produce a slurry. Given the microaerophilic nature of the sediment from these springs, the identification of anaerobic microbes within the Colour Peak Springs sediment [7,10,19], and the low levels of oxygen in the martian atmosphere [5], the enrichments were performed under anoxic conditions. Further to this, to assess the viability of members of the microbial community, rather than pure isolates, this growth series was performed using a total community approach, enriching from the environmental material. In triplicate, 1 mL of the slurry was transferred to the serum vials containing the different simulant materials. Abiotic controls with no Colour Peak Springs sediment were established in parallel. The enrichments were established under anoxic conditions with a headspace of H_2_/CO_2_ (80:20) at 1 bar pressure. However, whilst an estimate of ~1 bar atmosphere is feasible for the Noachian, it is possible it was either lower [20] or higher [21] during the Noachian–Hesperian transition, and this is therefore a caveat of this study. The vials were incubated for 28 days at 10 °C, representing the upper-range of temperatures observed at the springs on Axel Heiberg [6]. Microbial growth was controlled via cell counting. Cell counts were performed with a Leica DMRB microscope equipped with epifluorescence (Leica Microsystem, Bensheim, Germany), as previously described [22]. All enumerations were conducted with 50 fields of view counted per sample.

### 2.4. Characterisation of the Enriched Microbial Communities

DNA was extracted using a modified variant of the Griffiths technique [23] from 5 mL aliquots of each replicate from the endpoint of the enrichment. Nuclease free water was processed through the extraction as a negative extraction control. The V4-V5 region of the bacterial 16S rRNA gene was amplified by polymerase chain reaction using the primers com1 and com2 [24]. The PCR reaction mixture contained (per 25 µL): 1 × PCRBIO Ultra Polymerase red mix (PCR Biosystems, London, United Kingdom), 0.4 μM forward primer and 0.4 μM reverse primer. The PCR products were sequenced using the Illumina MiSeq Platform by Molecular Research LP, (Shallowater, TX, USA). The data were processed by Mr DNA using a customized pipeline [25,26]. All pair-end sequences were merged, chimeras removed and sequences less than 150 bp and/or with ambiguous base calls were removed. The sequences were clustered at 97% similarity and phylogeny was assigned using a curated database from GreenGenes, RDPII and NCBI [27]. Contaminant sequences identified in negative controls were eliminated from the datasets [28].

### 2.5. Analysis of Fluid Chemistry by ICP-OES 

To assess the impact of microbes on the NH fluid chemistries, the individual elements in the fluids from growth experiments were measured by inductively coupled plasma-optical emission spectroscopy (ICP–OES) using an Agilent 5110 model instrument, as previously described [7]. These fluids were taken from both the biotic and abiotic test groups at the end of the enrichment and filtered using 0.22 μm filters prior to analysis. 

## 3. Results

### 3.1. Impact of the Simulated Martian Fluid Chemistries on the Microbes of Colour Peak Springs 

**Figure 2:**
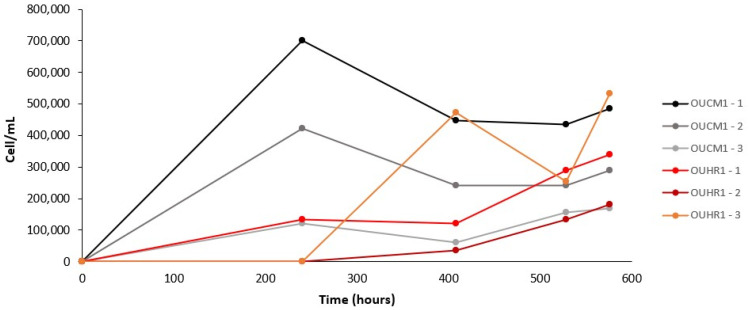
Cell counts of the Colour Peak Springs sediment enriched in simulated Noachian-Hesperian fluids with either the Gale Crater (OUCM-1) or Haematite Slope (OUHR-1). Second number in label represents replicate number.

Growth was observed in both test groups, the simulated NH fluid chemistry in combination with the basaltic OUCM-1 simulant and in combination with the Fe^3+^ enriched OUHR-1 simulant. However, there was highly variable growth observed within each test group (Figure 2). Initial cell counts based on 100 field of view counts following the inoculation were below the observation threshold. Growth was then most consistently observed in the OUCM-1 test group at 240 h (3/3 replicates) compared to the OUHR-1 test group (1/3). There was no consistent trend in abundance within the test groups with regards to growth, maximum cell abundances reached, or cell abundances at the end of the experiment, with each replicate exhibiting distinct growth dynamics. 

**Figure 3:**
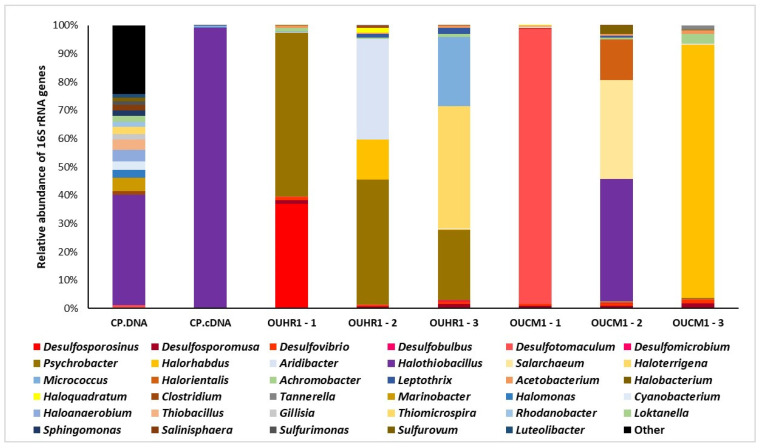
16S rRNA gene profiles of microbial communities enriched in simulated Noachian martian fluids, identified to genus level (>1% relative abundance). The 16S rRNA gene profiles of the nascent Colour Peak produced from DNA (CP.DNA) and cDNA (CP.cDNA) are from Macey et al., 2020. All genera pictured are present at >1% relative abundance. Sequences were revealed by amplicon sequencing of 16S rRNA gene amplicons retrieved by PCR from DNA extracted from the microbial communities enriched on the NH fluid chemistry and either the OUCM-1 or OUHR-1 simulant. The second number in the labels corresponds to replicate number.

Differences were also observed in community profiles of the enriched samples. Taxonomic assignment identified that most of the sequences belonged to the *Proteobacteria* and the archaeal class *Halobacteria* (Figure 3); each enriched community comprised of varying ratios of the archaeal class *Halobacteria*, halotolerant bacteria, and either sulfate-reducing or sulfur-oxidising bacteria. The microbial community of OUHR-1 1 was dominated by *Desulfosporosinus* (37%) and *Psychrobacter* (58%). *Psychrobacter* was also present in OUHR-1 2 and OUHR-1 3 (44% and 25%, respectively), with the community of OUHR-1 2 also comprised of *Aridibacter* (35%) and *Halorhabdus* (14%), whilst OUHR-1 3 was *Micrococcus* (24%) and *Haloterrigena* (43%). The most abundant genus in the microbial community of OUCM-1 1 was *Desulfotomaculum* (98%), whereas OUCM-1 3 was dominated by *Halorhabdus* (89%), and OUCM-1 2 was comprised of *Halothiobacillus* (43%), *Haloorientalis* (14%) and *Salarchaeum* (35%). 

### 3.2. Chemical Analysis of Simulated Martian Fluid Samples

Analysis of the fluids using ICP-OES after combination with the Colour Peak Springs sediment and either the OUCM-1 or OUHR-1 simulants for 28 days identified shifts in elemental abundance (Table 1). There were lower concentrations of Fe, Mn, Mo and Ba in the biotic test groups of the two fluid chemistries relative to the abiotic test group. In the biotic OUHR-1 test group, there was also a decrease in the concentration of P across the replicates that was not observed in the OUCM-1 test group. Relative to the abiotic test group, the pH declined significantly in the biotic test group in both the OUCM-1 (6.76 biotic, 7.28 abiotic) and OUHR-1 (8.58 biotic, 9.78 abiotic) fluid chemistries after 28 days of growth. 

## 4. Discussion

A range of techniques were applied to identify which microbes from Colour Peak, a martian analogue environment, were viable under simulated martian chemical conditions relevant to the waters that existed on the surface of Mars during the Noachian–Hesperian transition. Previous work [7] predicted that microbes from Colour Peak with sulfur-based metabolisms would be viable under these simulated martian chemical conditions, using analogue and modelling approaches. This paper takes this research further by experimentally testing the viability of the Colour Peak microbial community under laboratory-simulated martian chemical conditions. Growth was observed in the enrichments performed with the Colour Peak Springs sediment, confirming that this analogue environment contains microbes that are viable under simulated martian chemical conditions with an anaerobic atmosphere. The microbes from the microbial community of the sediment of Colour Peak that were shown to be capable of growth in one of the simulated martian chemistries of this experimental series can be broadly characterised as sulfate-reducing bacteria, sulfur-oxidising bacteria and heterotrophic halotolerant and halophilic microbes (both archaeal and bacterial). The impact of these simulated conditions on the growth, productivity, and diversity of microbes was observed, with the subsequent effect that microbes had on the fluid chemistries also examined. 

### 4.1. Variation in Viability of Colour Peak Microbial Community

Growth was observed in both fluid chemistries that evolved from interactions between the simulated NH fluids in combination with either the more basaltic simulant (OUCM-1) or the Fe^3+^ enriched simulant (OUHR-1). Of note is the observed variable enrichment of both bacteria and archaea in both test groups, with prior experiments that simulated martian fluid chemistries observing either an exclusive presence or a dominance by bacteria [29,30]. The archaeal diversity was composed of halophilic archaea (class *Halobacteria*). The observed high levels of enrichment of the archaeal class *Halobacteria* in this instance could be due to the use of different inoculum sources or the chemistry of the NH fluid in combination with the simulant regolith chemistries used in this study representing chemical environments that archaea were better able to exploit. Magnesium also play an important role in supporting the growth of Halobacteria, with several genera requiring specific concentrations to be capable of growth [31]. Unlike most halophilic or halotolerant bacteria, halophilic archaea in general favour maintaining osmotic balance with the external environment by accepting salt ions into their cells [32]. This strategy requires proteomic structural adaptation and has led to specific requirements by halophilic archaea for Na, K and Cl ions [33], all of which were present in appreciable quantities in the NH fluids. It is worth highlighting that the relative success of, and dominance of, a variety of natural environments by either bacteria or archaea is a long-standing debate within microbiology. Some authors point to an apparent advantage of archaea in energetically challenging environments [34], while others have noted an apparent competitive disadvantage with increased N concentrations [35]. Thus, it is likely that a complex interaction of several factors is at play.

Each enrichment also exhibited reductions in diversity from the starting inoculum, each of the enrichments dominated by three or fewer genera (Figure 3). The initial Colour Peak community was comprised predominantly of *Proteobacteria* (74–78% relative abundance), specifically *Gammaproteobacteria* (60% relative abundance of the community), represented by genera associated with sulfur oxidation or shown to contain species capable of sulfur oxidation (*Halothiobacillus, Thiobacillus*, *Thiomicrosospira*, *Halomonas*, *Marinobacter* and *Salinisphaera*). Additional community members were *Bacteroidetes* (8%), *Firmicutes* (6–9%), *Betaproteobacteria* (4–6%), *Epsilonbacteraetota* (2–3%) and *Cyanobacteria* (3%). Sulfate-reducing bacteria were also detected in the 16S rRNA gene profile, representing 1.5–2% relative abundance, with the genera *Desulfocapsa* and *Desulfuromonas* detected at >0.1% relative abundance. In the 16S rRNA gene profile generated from cDNA produced from RNA extracted from the sediment, no sulfate-reducing bacteria were detected and *Halothiobacillus* was present at 98% relative abundance [7]. Except for OUCM-1 2, in which *Halothiobacillus* was the most abundant genus, there was minimal conservation with regards to the nascent Colour Peak communities and the enriched communities. This was also observed between and within the basaltic and Fe^3+^ enriched test groups, with each replicate enriching a taxonomically distinct community. This result shows that that the Colour Peak sediment contained numerous taxa that were capable of growth under simulated martian chemical environments, with most of these enriched microbes representing the rare microbiome (present at low or undetected abundance) in the unenriched community. It is possible that variation between the replicates was the result of successional waves that occurred in some of the enrichments and had not occurred in others [36]. Some of the enrichments were dominated by clades associated with autotrophy, which could be tentatively classified as primary producers (sulfate-reducing and sulfur-oxidising bacteria), with other bacteria and archaea also present. Other communities were dominated by clades associated with heterotrophy. Given the low level of organic carbon within the simulant material, sediment, and the fluid chemistry, this would suggest that this diversity might represent syntrophic microbes. Other possible factors may be interactions at the community level, such as syntrophy, variation in the starting community supplied by the inoculum and stochastic variation in growth [37,38].

The observed difference of the enriched communities from the unenriched sediment could be a result of enhanced competitive efficiency of the enriched genera in the supplied fluid and headspace chemistries [39,40]. Some strains of bacteria are also shown to thrive under enrichment conditions, resulting in the bias that is often observed in cultivation-dependant studies relative to characterisation of nascent communities [41,42]. Alternatively, it is also possible that some members of the original and unenriched community were unable to survive in the simulated martian chemistries; however, this is shown to not be the case for *Halothiobacillus*, as it is shown to persist in one of the replicates. 

Whilst taxonomically distinct, there is also some degree of consistency with regards to the functional diversity of the enrichments, with the presence of a minimum of 1.5% of the community represented by bacteria that can be putatively identified as sulfur cycling, either sulfur-oxidising (*Halothiobacillus*) or sulfate-reducing bacteria (*Desulfosporosinus*, *Desulfosporomusa*, *Desulfovibrio*, *Desulfobulbus*, *Desulfotomaculum*, *Desulfomicrobium*). This putative identification is derived from the high levels of taxonomic conservation with regards to the presence of these metabolisms within the identified taxa [43,44,45]. In five out of the six enrichments, sulfate-reducing bacteria increase in relative abundance relative to the nascent community. However, in one of the communities the dominant sulfur-oxidising bacterium identified in the 16S rRNA gene profile of the nascent Colour Peak Springs sediment remains the most abundant genus. Given this observed enrichment of sulfur-oxidising bacteria and the additional heterotrophic microbes, this supports the proposed viability of a sulfur oxidation driven carbon cycle under martian conditions and their possible role in enabling the viability of additional heterotrophic bacteria [7]. 

### 4.2. Microbial Influence on Environmental Chemistry

The observation of a reduction in specific elements in the biotic test groups relative to the abiotic is potentially a result of sequestration of these elements in either cells or precipitates that formed in this test group. Inferring the past presence of microbial communities on ancient Mars may rely in part on understanding how such communities would have influenced their surrounding environment. These findings showed that there were some limited alterations in fluid chemistry that were exclusive to the different test groups (NH fluid and OUCM-1 and NH fluid and OUHR-1). This variation between test groups supports the idea that the potential impact of a microbiome on an environment could vary between environments with different lithologies. This is important to consider with regards to potential biosignature identification between geographically or geologically distinct points on Mars (e.g., Gale Crater and Haematite Slope [15,16]). However, constraining parameters in which biological impacts on fluid chemistry could result in biosignature formation also requires the characterisation of shifts in chemistry that arise under abiotic conditions over geological timescales. This is important as microbes can enhance the rate at which minerals or volatiles form but are not solely responsible for their formation, meaning non-biological reasons for their presence cannot be discounted (i.e., sulfate-reducing bacteria and the formation of iron containing minerals and hydrogen sulfide in anoxic fluids [46,47]). 

It is also possible that additional analyses might be capable of identifying unambiguous biosignatures arising from the presence of the microbes enriched in this study. Some possible biological impacts unconstrained to specific functional or taxonomic groups include depletion of ^13^C due to isotope fractionation [48], the entombment of cells, lipids or other organic matter [49,50,51], or shifts in concentration of specific trace elements [49]. In terms of taxonomically restricted biosignatures, halophiles, sulfate reducers and sulfur oxidisers all contain specific pigments, protein subunits and lipids that could be preserved within rocks and spectrally detected (e.g., pterin-containing protein subunits in sulfur oxidisers and Bacterioruberin) [12,14,52]. Sulfate reduction is also well documented to result in isotope fractionation, including in Mars analogue environments, resulting in the depletion of ^34^S within metabolic products, with a similar shift shown to occur less consistently as a result of sulfur oxidation [53,54].

## 5. Conclusions

Identifying martian analogues and developing an understanding of the interaction between their biota and martian chemical systems through simulation studies is key to developing hypotheses with regards to habitability, the metabolisms that could have been viable within these environments and the possible associated biosignature formation. This work also shows that there were exclusive alterations in fluid chemistry between the different test groups (Noachian and OUCM-1 and Noachian and OUHR-1). This study shows that microbes from two distinct analogue environments are capable of growth in a range of aqueous chemistries simulating water that might have existed on mars and provides experimental support for previous suggestions of sulfur cycling as a viable metabolism in chemistries that may have existed during the Noachian–Hesperian transition.

## Figures and Tables

**Table 1:** ICP-OES analysis of NH fluid and either OUCM-1 or OUHR-1 simulant material under either abiotic conditions or combined with sediment from the Colour Peak Springs sediment after 28 days of incubation. Values are in mM.

Environment	OUCM-1	OUCM-1	OUHR-1	OUHR-1
Test group	Biotic	Abiotic	Biotic	Abiotic
As	1.8 (0.01)	0.36 (0.017)	0.015 (0.015)	0.16 (0.024)
Ba	0.062 (0.005)	0.18 (0.002)	0.051 (0.001)	BD
Ca	720 (3.1)	260 (1.3)	1800 (6.2)	2000 (18)
Fe	2.7 (0.25)	71 (17)	0.13 (0.072)	110 (4.8)
K	160 (0.81)	150 (0.99)	180 (0.97)	180 (1.4)
Mg	130 (1.2)	75 (0.56)	310 (0.32)	95 (0.85)
Mn	2.9 (0.023)	11 (0.14)	9.3 (0.056)	45 (0.32)
Na	890 (4.7)	820 (2.6)	910 (3.8)	830 (3.9)
Nd	BD	BD	0.006 (0.003)	0.007 (0.003)
P	49 (49)	230 (53)	260 (61)	250 (79)
S	710 (2.2)	390 (0.61)	3500 (11)	3000 (26)
Sb	0.31 (0.006)	0.22 (0.004)	0.18 (0.003)	0.45 (0.015)
Si	170 (0.49)	99 (0.51)	190 (0.69)	180 (3.7)
Sn	0.38 (0.053)	0	0.51 (0.095)	0.479 (0.016)

BD—Below detection. Numbers in brackets represent Standard Deviation of measurements done in triplicate.

## Data Availability

Amplicon sequence data generated in this study were deposited to NCBI GenBank under numbers SRX9907173-SRX9907178.
